# Extreme Multiple Reticulate Origins of the *Pteris cadieri* Complex (Pteridaceae)

**DOI:** 10.3390/ijms13044523

**Published:** 2012-04-10

**Authors:** Yi-Shan Chao, Shi-Yong Dong, Yu-Chung Chiang, Ho-Yih Liu, Wen-Liang Chiou

**Affiliations:** 1 Division of Botanical Garden, Taiwan Forestry Research Institute, Taipei 10006, Taiwan; E-Mail: pteridaceae@gmail.com; 2 South China Botanical Garden, Chinese Academy of Sciences, Guangzhou 510650, China; E-Mail: dongshiyong@scib.ac.cn; 3 Department of Biological Sciences, National Sun Yat-Sen University, Kaohsiung 80424, Taiwan; E-Mail: yuchung@mail.nsysu.edu.tw

**Keywords:** apogamy, flow cytometry, hybridization, *PgiC*, polyploidy, *Pteris*, reticulate evolution, species complex

## Abstract

The *Pteris cadieri* complex displays extensive morphological variation and seems to have originated through hybridization. However, the members of this complex reproduce by apogamy, which usually limits genetic variation. To evaluate the hypotheses of hybrid origins, the pattern of evolution in this species complex is reconstructed. Multiple methodologies were used. Diploids, triploids, and tetraploids were identified by chromosome counts and flow cytometry. Nuclear DNA markers (cytosolic phosphoglucose isomerase gene, *PgiC*) were used, together with chloroplast DNA markers (*atpB-rbcL* spacer and *rbcL* gene) to infer the biparental and maternal lineages of the *Pteris cadieri* complex. The three cpDNA haplotype groups and five *PgiC* alleles found in this study indicate that the evolution of the *Pteris cadieri* complex has been extremely reticulate. Up to 11 taxa belonging to eight morphs were identified. By comparing genetic variation in the *Pteris cadieri* in two independent areas, Hainan and Taiwan, we inferred that hybridization has occurred independently in different areas. Furthermore, we found evidence for phenological divergence (evergreen and deciduous) within Taiwan. We propose that the *Pteris cadieri* complex originated from different genetic lineages through multiple hybridizations in different geographical areas, leading to its present morphological diversity.

## 1. Introduction

Plants arising from hybridization usually have varied morphologies, which usually exhibit a gradual cline [1,2]. In some cases, the hybrids can cross with their parents and result in offspring with diverse genetic characters [3–5]. The delimitation of hybrid offsprings and the parental species becomes gradually indistinguishable. Hybridization has been demonstrated in ferns with great morphological variation, including *Asplenium* [6], *Athyrium* [7], *Polystichum* [8], *Cheilanthes* [9,10], and the *Vandenboschia radicans* complex [11]. Although most hybrid fern species seem to have high levels of genetic variation, apogamous ferns, usually the result of hybridization, have limited genetic variation [12]. Apogamy is a special form of asexual reproduction in ferns and provides a means for overcoming the inability of non-homologous chromosomes to pair in hybrid ferns [13–15]. The parents and offspring of apogamous ferns have almost identical genetic characters and thus express the same morphologies. As a result, the mechanism of apogamy creates reproductive barriers that prevent gene flow among closely-related taxa, facilitating sympatric speciation.

The *Pteris cadieri* complex is widely distributed in eastern and southern Asia. The scale of morphological variation in *P. cadieri*, whose fronds range from simply pinnate to bipinnatifid, has led several authors to treat *P. cadieri* as several different species, including *P. cadieri* Christ, *P. dimorpha* Copel., *P. grevilleana* Wall. *ex* J. Agardh, *P. hainanensis* Ching, *P. plumbea* Christ, and *P*. × *sintenensis* (Masam.) Kuo [16–19]. The morphology of these taxa overlapped to such an extent that it was difficult to identify each species unambiguously, leading some authors to treat them as synonyms of *P. cadieri* [16,19,20]. The significant morphological variation noted in the description of *P. cadieri* lead to the hypothesis that it arose through hybridization [21–23]. *Pteris grevilleana*, with bipinnatifid fronds, was thought to be a parent of *P. cadieri* [22–24]. Because of the apogamy of *P. cadieri* and *P. grevilleana*, both species are thought to have arisen from hybridization. However, *P. grevileana* should not be a parent of *P. cadieri* [25]. Their exact relationships remain unclear. On the other hand, the *P. cadieri* complex is apogamous but has distinctly great morphological variation. This complex could arise from complicated evolutionary events.

Recently, several hypotheses have described how species could be derived from multiple origins by a number of mechanisms [26], with recurrent hybridization events and polyploidy considered most important [27–29]. Diverse cytotypes and genetic lineages are indicative of multiple hybridization and polyploidy [30–32]. Because each population/taxon could arise independently, geographical patterns could provide valuable insights into historical processes [33–35].

Nucleotide variation in plastid genes has proven a powerful tool for reconstructing plant phylogeny. Nuclear DNA data can provide bi-parental information about hybrids. In ferns, however, few nuclear markers have been developed, and some have disconcerting limitations. For example, internal transcribed spacers (ITS), popular sequences for phylogenetic inference, are of limited utility in ferns due to high homoplasy, bias of one parent, and difficult sequencing caused by secondary structure [36–38]. Single-copy genes are more easily analyzed than low-copy genes. Among gene markers commonly used in ferns, *leafy* and *gapCp* were reported to be multiple-copy genes in some species [10,39,40], therefore, cytosolic phosphoglucose isomerase (*PgiC*) is considered the most suitable single-copy gene [41,42].

To investigate the *Pteris cadieri* species complex, with its great morphological variation, we collected plants with as many different morphologies as possible. However, this complex is widely distributed in eastern and southern Asia, so we could not obtain samples across its entire range for this study. For this study, we collected samples from two independent geographic regions, the islands of Hainan and Taiwan. We sampled extensively and tried to obtain samples of every morphological variant observed in the distinct populations on each island. We explored the evolutionary origins of the *P. cadieri* complex using multiple methodologies. To detect the presence of polyploidy, cytotypes were determined using cytological analyses. To detect hybrid origins, molecular analyses, including chloroplast (*atpB-rbcL* spacer and *rbcL* gene) and nuclear marker (*PgiC* gene) data, were used to make inferences about the maternal and paternal genetic lineages. Based on these analyses, we discuss the relationship between morphological variation, ploidy, and recurrent origins in the *P. cadieri* complex.

## 2. Results and Discussion

### 2.1. Results

#### 2.1.1. Morphological Discrimination

**Figure 1:**
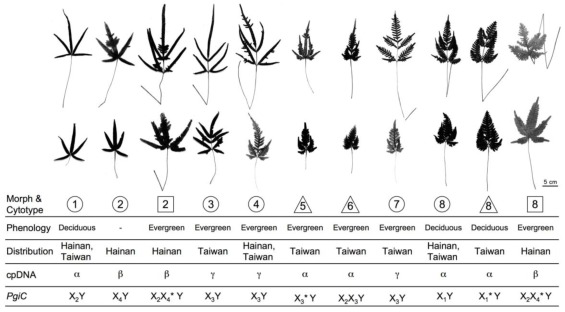
Morphologies and key characters of the eight morphs in the *Pteris cadieri* complex. Two leaves of each morph are shown. The upper leaf is fertile, and the lower leaf is sterile. The key characters include cytotype, phenology (evergreen or deciduous), geographic distribution, chloroplast haplotype, and *PgiC* genotype. An asterisk * represents an uncertain allele, e.g., genotype X_1_*Y means X_1_X_1_Y or X_1_YY. Symbols indicate cytotypes: ○ diploid; ▵ triploid; □ tetraploid. The number in each symbol indicates the morph.

Samples were collected of each morphological variant in the *Pteris cadieri* complex from six populations in Hainan and 21 populations in Taiwan. Based on our careful and extensive searches of both islands we think we obtained samples from each population on Hainan and Taiwan [43,44]. Voucher specimens were deposited in the herbarium (TAIF) of the Taiwan Forest Research Institute (Supplementary S1). Altogether, eight morphological types were identified (morph 1 to morph 8), including simply pinnate, irregularly bipinnatifid, and regularly bipinnatifid. The morphological characters of each morph are summarized in Table 1 (see Figure 1 photos). Compared to the type specimens of related taxa, several morphs corresponded to previously described taxa: morph 1 was *P. cadieri*, morph 2 was *P. hainanensis*, morph 3 was *P. plumbea*, morph 4 was *P. dimorpha*, and morph 8 was *P. grevilleana*. However, morphs 5, 6, and 7 had no corresponding scientific names.

#### 2.1.2. Cytotypes, Phenology, and Reproductive Systems

**Figure 2:**
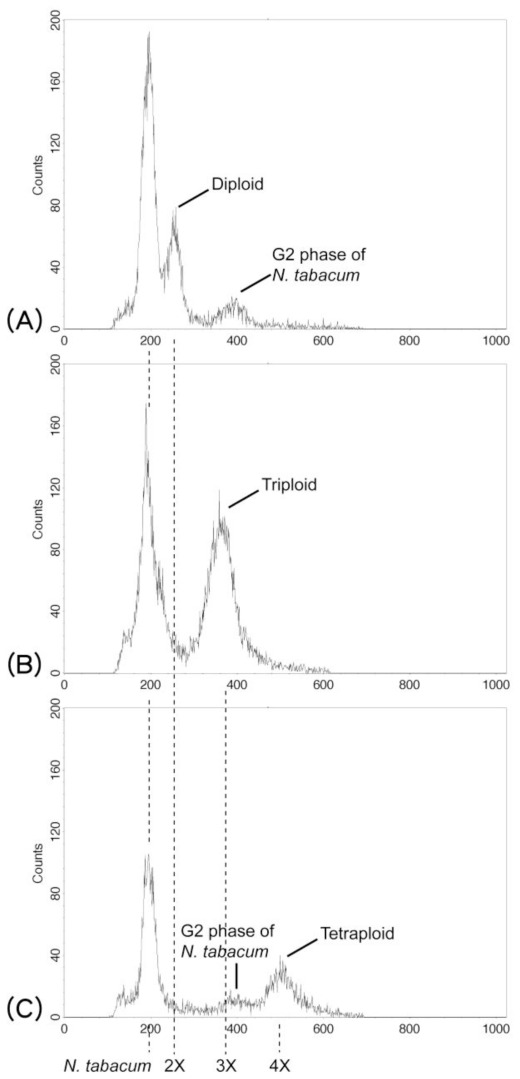
Examples of the *Pteris cadieri* complex cytotype (2X, 3X, 4X) determination by flow cytometry. The horizontal axis indicates fluorescence intensity. *Nicotiana tabacum* was used as a calibration standard. There is a ratio 2:3:4 among the values of diploid, triploid and tetraploid. The vertical axis indicates the number of cells.

Using flow cytometry, the cytotypes of each plant in the *P. cadieri* complex were determined. *Nicotiana tabacum* L. “Xanthi” (4X = 20.08 pg; [45]) was used as a calibration standard to infer the genome size of the *P. cadieri* complex. We compared cytometric data for *N. tabacum* with those for diploid, triploid, and tetraploid members of the *P. cadieri* complex (Figure 2). The *P. cadieri* complex/*N. tabacum* genome ratios were 1.2, 1.8, or 2.4. Based on the genome size of *N. tabacum* and plants with known cytotypes (diploid and triploid, [25]), the genome size of diploid and triploid plants of the *P. cadieri* complex was approximately 24 pg (=20.08 pg × 1.2) and 36 pg (=20.08 pg × 1.8), respectively. We inferred that *P. cadieri* complex plants with a genome size of approximately 48 pg (=20.08 pg × 2.4) were tetraploid. The ploidy level of each morph was identified: morphs 1, 3, 4, and 7 were diploid; morphs 5 and 6 were triploid; morph 2 was diploid or tetraploid; morph 8 was diploid, triploid, or tetraploid. Therefore, the *P. cadieri* complex is comprised of 11 taxa altogether (Figure 1).

Ferns were cultivated in the Taipei Botanical Garden greenhouse for one to five years. Although all plants were collected at altitudes below 1000 m, their phenological characters were different. Morphs 3, 4, 5, 6, 7, and tetraploid morphs 2 and 8, were evergreen, whereas morph 1 and diploid and triploid morph 8 were deciduous in winter. Tetraploid morph 2 (from Hainan) were not studied because too few plants were cultivated.

The number of spores per sporangium was counted for each plant, except for a few plants that lacked fertile blades. All fertile plants had sporangia with 32 spores. A few sporangia had only 16 or 28 spores. Since no 64-spore sporangia were found, all the observed plants were determined to be apogamous.

#### 2.1.3. Phylogenetic Resolution of Chloroplast DNA Data

**Figure 3:**
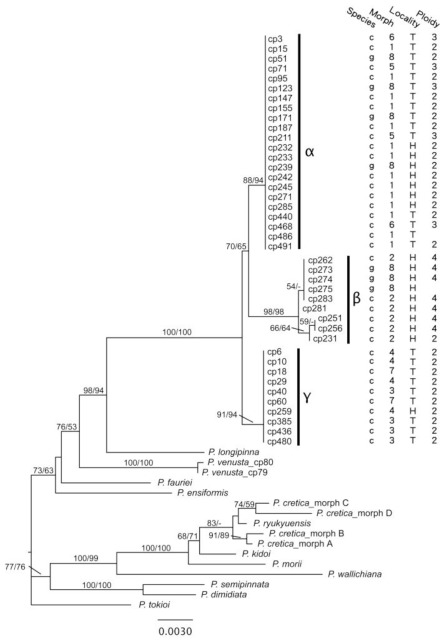
Chloroplast DNA tree for the *Pteris cadieri* complex and other *Pteris* species as determined by ML analyses. Based on the *atpB-rbcL* spacer and *rbcL* gene, plants of the *P. cadieri* complex, shown by specimen codes, were monophyletic, distinctly separate from the other *Pteris* species, and divided into three haplotype groups, α, β, and, γ. Species (c, *P. cadieri*; g, *P. grevilleana*), morph (1–8), locality (H, Hainan; T, Taiwan), and ploidy level (2, diploid; 3, triploid; 4, tetraploid) are shown. Numbers above the branches are ML/MP bootstrap values.

We performed the following phylogenetic analyses using the combined sequences of the two regions, including the *atpB*-*rbcL* spacer and *rbcL* gene (primers in Table 2). The ILD test did not show significant incongruence (*P* value = 0.289). General features of the DNA regions are summarized in Table 3. For the combined cpDNA dataset, the 50% majority rule consensus MP tree and the ML trees had identical topology (the ML tree is shown in Figure 3). The *P. cadieri* complex formed a well supported, monophyletic group distinct from the other *Pteris* species included in the analysis. The closest outgroup species was *P. longipinna*. The chloroplast haplotypes of the *P. cadieri* complex were assigned to three, moderately- to well-supported clades: haplotype groups α, β, and γ. *Pteris cadieri* had haplotype groups α, β, and γ, whereas *P. grevilleana* had haplotype groups α and β. Most morphs had a single haplotype group, except morph 8, which had two haplotype groups. Diploid and triploid morph 8 belonged to haplotype α, and tetraploid morph 8 belonged to haplotype β. Ploidy levels are shown on the chloroplast ML tree (Figure 3).

#### 2.1.4. Phylogenetic Resolution of *PgiC* Gene Data

**Figure 4:**
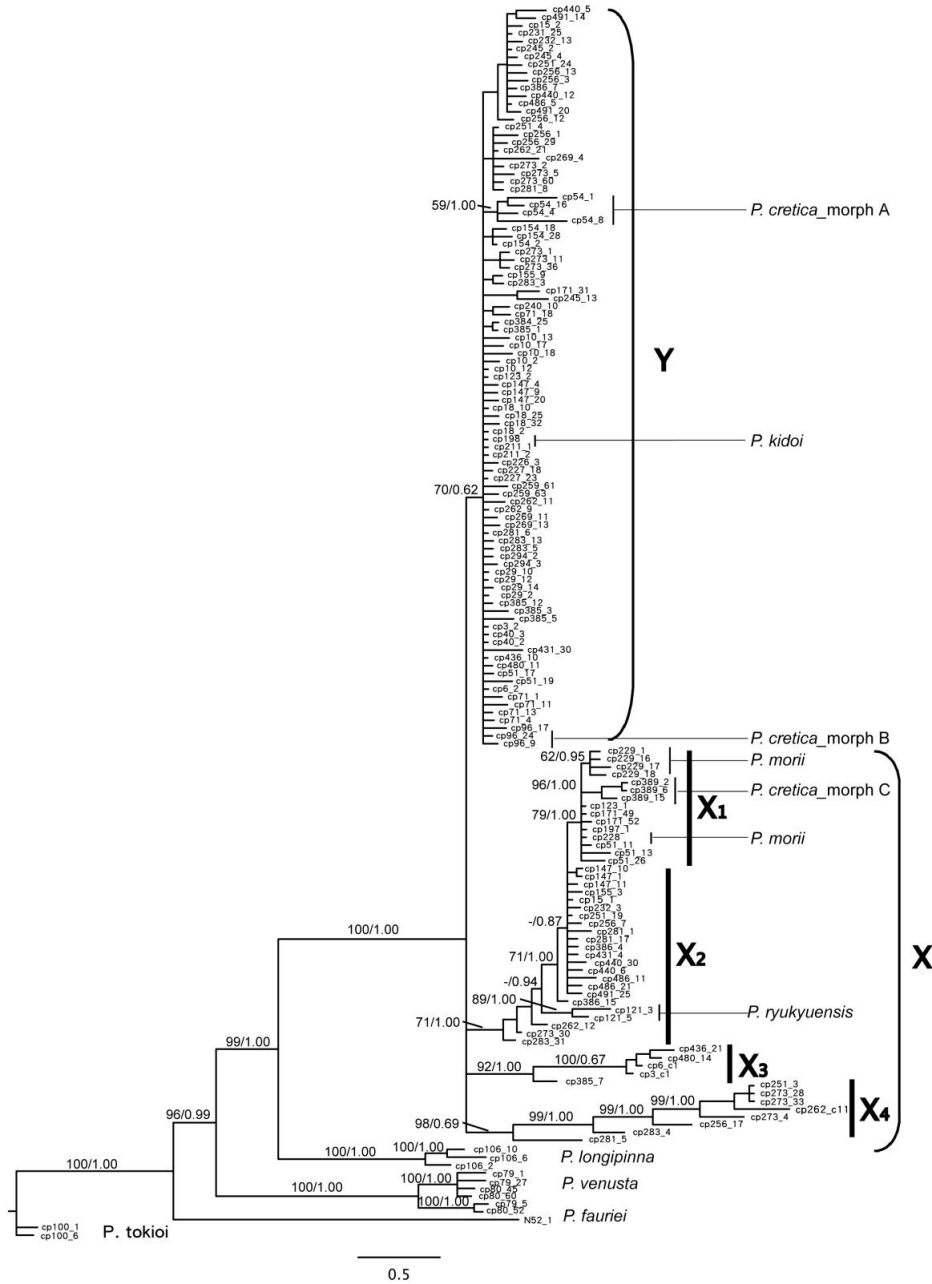
Nuclear DNA tree assessed by Bayesian inference for *Pteris cadieri* complex and other *Pteris* species. Based on the cytosolic phosphoglucose isomerase gene (*PgiC*) (primers 15PF and 17R), each clone is identified to alleles X_1_, X_2_, X_3_, X_4_ or Y. The number following the specimen code indicates the cloning sample (see Supplementary S1). If a specimen code is not followed by a cloning sample number, the sample was sequenced directly. Numbers above branches are MP bootstrap supporting/posterior probability (PP) (>50; >50%) values.

Existing primers for the *PgiC* gene in ferns do not work well in some taxa [10,11], therefore, we developed new primers for this nuclear marker in *Pteris*. Excluding the clones with repeated sequences, a total of 158 sequences of the *PgiC* gene were analyzed. After removing an ambiguously aligned region within intron 15, the unaligned length of long nuclear *PgiC* gene sequences, by means of primers 15PF and 17R, ranged from 1431 to 1705 bp. The aligned *PgiC* gene, 1744 bp, was analyzed directly using Bayesian inference. Bayesian tree and posterior probabilities are shown in Figure 4. Furthermore, for MP analysis, indels were coded and 50 binary characters produced to combine with the aligned *PgiC* gene. MP tree statistics are summarized in Table 3. Because the strict consensus tree from MP analysis (not shown) was similar to the inferred Bayesian tree, the corresponding bootstrap values were marked on the Bayesian tree (Figure 4).

Because of the distinctly morphological and phylogenetic difference, *P. fauriei*, *P. longipinna*, *P. tokioi*, and *P. venusta*, not involving in the *P. cadieri* complex, were used as outgroups, and their positions were consistent with the cpDNA tree. All clones of the *P. cadieri* complex were united in a well supported clade, including groups X and Y (Figure 4). Clones in group Y, defined as allele Y, were moderately supported and had a 15 bp synapomorphic deletion between exons 15 and 16 of *PgiC* compared to group X. Group X was comprised of subsclades X_1_, X_2_, X_3_, and X_4_, defined as alleles X_1_, X_2_, X_3_, and X_4_.

To avoid PCR bias [50], forward primers 15PFX or 15PFY, and reverse primer 17R, were used for additional sampling. These shorter sequences were combined with longer sequences from primers 15PF, and 17R and coded the indels (see section 3.4. Phylogenetic analysis). After removing clones with identical sequences, a total of 191 sequences of the *PgiC* gene were analyzed. The MP tree statistics are in Table 3. The results were used to determine the *PgiC* genotype of each plant (Supplementary S1 and Figure S2). The pattern of grouping was the same as the pattern from longer sequences (Figure 4). However, the topology had lower branch supporting values than in Figure 4. This is likely the result of having fewer parsimony informative characters.

Alleles of each individual in the *P. cadieri* complex fell into two or three clades of the *PgiC* gene tree: one clade was always allele Y and the others were alleles X_1_, X_2_, X_3_, or X_4_. Diploids had two alleles (e.g., morph 1 was X_2_ and Y), and triploids had two or three alleles (e.g., morph 5 was X_3_ and Y; morph 6 was X_2_, X_3_, and Y). The number of alleles was less than the ploidy level in some triploid and tetraploid plants. Based on sequences from a large number of clones, we inferred that this discrepancy could be caused by allele dosage; *i.e.*, one of the alleles was present multiple times in a polyploid genome. Considering the position of alleles in the *PgiC* gene tree, cytotypes, and allele dosages, the genotypes of eight morphs were determined (Figure 1, Supplementary S1). Genotypes with an asterisk * indicate uncertain alleles [11]. For example, the genotype of morph 5, with the *PgiC* alleles X_3_ and Y, is shown as X_3_*Y because it could be X_3_X_3_Y or X_3_YY.

Although the *PgiC* genotype of each morph was determined, each genotype was not unique to a specific morph. For example, morphs 3, 4, and 7 had different morphologies but had the genotypes, X_3_Y.

Morphs with the same cpDNA haplotype group may have different *PgiC* genotypes, and *vice versa*. For example, morphs 1 and 8 share haplotype group α, but have *PgiC* genotypes X_2_Y and X_1_*Y respectively. In contrast, some morphs shared *PgiC* genotypes but had different cpDNA haplotypes. For example, both morphs 4 and 5 have *PgiC* X_3_ and Y alleles, but they have cpDNA haplotype groups γ and α, respectively. Such taxa have different maternal or paternal lineages.

#### 2.1.5. Divergence in Different Geographical Areas and Phenology

**Figure 5:**
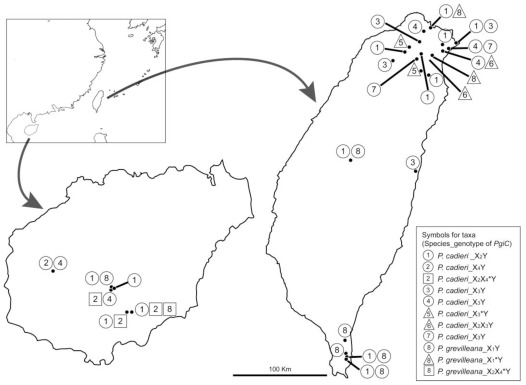
Geographic distribution of the eight morphs in the *Pteris cadieri* complex from Hainan and Taiwan. Populations are shown as dots. There were six populations in Hainan and 21 populations in Taiwan. Symbols indicate cytotypes: ○ diploid; ▵ triploid; □ tetraploid. The number in each symbol indicates the morph.

The geographic distribution of the eight morphs in the *P. cadieri* complex from Hainan and Taiwan are shown in Figure 5. A total of 27 populations, six in Hainan and 21 in Taiwan, were investigated. Morphs 1, 2, 4, and 8 occurred in Hainan, while morphs 1, 3, 4, 5, 6, 7, and 8 were found in Taiwan. Morphs 1 and 4, and the diploid form of morph 8 occurred in both Hainan and Taiwan. Morph 2 and the tetraploid form of morph 8 were restricted to Hainan, while morphs 3, 5, 6, 7, and the triploid form of morph 8 were found only in Taiwan. Genetic variation in *P. cadieri* complex cpDNA was greater in Hainan (α, β, and γ) than in Taiwan (α, γ). For the *PgiC* gene, the only difference between taxa in Hainan and Taiwan was that allele X_4_ was restricted to Hainan.

Tetraploids (in morphs 2 and 8) were found only in Hainan, and triploids (morphs 5, 6 and 8) occurred only in Taiwan. Because both morphs 2 and 8 occur only in Hainan, tetraploids may be associated with *PgiC* allele X_4_. In contrast, although the *PgiC* alleles X_2_, X_3_, and Y were found in Hainan and Taiwan, triploids (X_3_*Y, X_2_X_3_Y and X_1_*Y) were found only in Taiwan.

### 2.2. Discussion

#### 2.2.1. Hybrid Origin

The *Pteris cadieri* complex is apogamous, which is usually thought to be with low genetic variation. However, it exhibits distinct morphological and phenological variation. Based on bi-parentally nuclear DNA and maternally inherited cpDNA [51], maternal and paternal lineages were inferred in this study. Our results suggest that the *P. cadieri* complex arose from multiple, recurrent hybridizations which may be responsible for the wide range of morphologies in this apogamous taxon. Furthermore, due to apogamy, the heterozygosity of *PgiC* gene in hybrids is preserved; each lineage is independent and free from confusion of recombination.

In the *PgiC* gene tree, *P. longipinna*, *P. venusta*, *P. fauriei*, and *P. tokioi* had its own independent clade. The other species, excluding the *P. cadieri* complex, belonged to a single clade, indicating that their genotypes are XX or YY (morphs A, B, and C of *P. cretica* L. were regarded as different taxa; Supplementary S1). This indicates that the genetic variation between the two clades is greater than intraspecific genetic diversity within each clade. Because the alleles of each taxon in the *P. cadieri* complex separated into two or three different clades, taxa in this complex were probably derived from several distinct species; that is, they originated through hybridization. In addition, apogamy is characteristic of species in the *P. cadieri* complex [25,52], a trait found in other ferns thought to have arisen through hybridization [13,14,52–54].

*Pteris grevilleana* (morph 8 in this study) was proposed as a probable ancestor of *P. cadieri* [23]. However, evidence from the *PgiC* gene (Figure 4 and Figure S2) indicates that *P. grevilleana* was neither a maternal nor a paternal parent of other species in the *P. cadieri* complex. Because *P. grevilleana*, like other taxa in the *P. cadieri* complex, has two or more distinctly different alleles, it probably also arose through hybridization.

#### 2.2.2. Parental Species?

Phylogenetic analyses of cpDNA revealed that the taxa in the *P. cadieri* complex (including *P. grevilleana*) were distinct from other *Pteris* species (Figure 3). Thus, species previously proposed as potential parents of *Pteris* species with linear pinnae, including *P. cretica* L., *P. ensiformis* Burm., *P. kidoi* Sa. Kurata, *P. ryukyuensis* Tagawa, *P. morii* Masam., and *P. semipinnata* L. [18,22–24,55], could not be direct maternal parents of taxa in the *P. cadieri* complex. Moreover, the three haplotype groups of cpDNA phylogenetic tree indicate three absolutely different maternal elements. Without observing other *Pteris* species, the maternal elements of the *P. cadieri* complex probably came from the taxa in the complex itself or from extinct species.

Given that there are five *PgiC* alleles X_1_, X_2_, X_3_, X_4_, and Y, there could be five diploid ancestral taxa, with the genotypes X_1_X_1_, X_2_X_2_, X_3_X_3_, X_4_X_4_ and YY involved in the origin of the *P. cadieri* complex. Considering alleles X_1_ and X_2_, it is likely that *P. cretica*, *P. kidoi*, *P. ryukyuensis*, and *P. morii* represent the paternal progenitors or are closely allied to the paternal progenitors of the *P. cadieri* complex. Thus, allele X_1_ is the maternal element of diploid morph 8 and triploid morph 8, and allele X_2_ is the maternal element of morphs 1 and 6.

Alleles X_3_ and X_4_ appeared only in the *P. cadieri* complex and not in other *Pteris* species. Comparing the nuclear DNA and cpDNA trees, except for morphs 5 and 6, taxa with the alleles X_3_ or X_4_ correspond completely to groups γ or β, respectively. Although chloroplast and nuclear genomes evolve independently, this correspondence suggests that the maternal element of morphs 3, 4, and 7 is allele X_3_, and the maternal element of diploid morph 2, tetraploid morph 2, and tetraploid morph 8 is X_4_. Based on current data, it is difficult to further infer the paternal or maternal lineage of morph 5. Thus, some parent species of the *P. cadieri* complex still have not been identified. It is possible that the ancestral species are extinct or have yet to be discovered.

#### 2.2.3. Reticulate Evolution of the *Pteris cadieri* Complex

**Figure 6:**
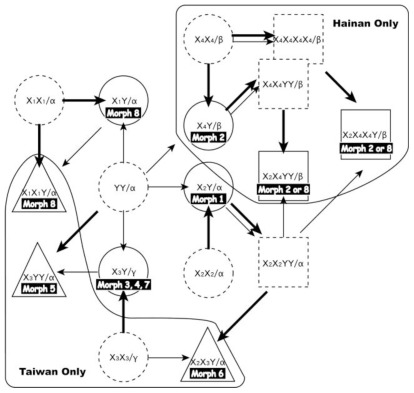
Inferred reticulate relationship of the *Pteris cadieri* complex. The most parsimonious scenario is exhibited. Symbols indicate cytotypes: ○ diploid; ▵ triploid; □ tetraploid. Symbols with a dotted outline indicate missing taxa. Maternal (bold line) and paternal (fine line) lineages were determined by comparing cpDNA and *PgiC* gene phylogenies. Taxa restricted to Hainan or Taiwan are indicated.

Based on the cpDNA and nuclear DNA phylogenetic data, reticulate patterns of the *Pteris cadieri* complex were constructed, including the inferred paternal and maternal lineages (Figure 6). Using a “diploids-first” strategy [56], five diploid, sexual taxa, with the genotypes X_1_X_1_, X_2_X_2_, X_3_X_3_, X_4_X_4_ and YY of *PgiC* gene, are proposed as the progenitors of the *P. cadieri* complex. Furthermore, four sexual tetraploids were presumed, including X_4_X_4_X_4_X_4_, X_2_X_2_YY, and X_4_X_4_YY. They could arise from diploidy of X_4_X_4_, X_2_Y, and X_4_Y, respectively (Figure 6). Genotypes X_2_X_2_YY, and X_4_X_4_YY could also arise from genome addition as hybridization, such as X_2_X_2_ × YY, and X_4_X_4_ × YY, respectively (not shown).

It is then straightforward to infer the origin of the diploid apogamous taxa in the *P. cadieri* complex. For example, morph 1 could be derived from hybridization between the sexual parental taxa YY and X_2_X_2_. Apogamous triploids could arise from a cross between sexual diploid and sexual tetraploid species or between apogamous diploids (unreduced, diploid gametes, functionally male) and sexual diploids [53,54,57,58]. For example, morph 6 with the genotype X_2_X_3_Y, could arise via X_2_X_2_ × X_3_X_3_YY, X_3_X_3_ × X_2_X_2_YY, or X_2_X_2_ × X_3_Y, where a diploid taxon with X_3_Y is presumed to be apogamous and functionally male. Because allele dosages were not determined in this study, inferences about the origin of other triploid and tetraploid taxa are difficult to make. For example, the *PgiC* genotype of morph 5 is either X_3_X_3_Y or X_3_YY, and each genotype has a different evolutionary history. Inferring evolutionary histories becomes even more complicated in the tetraploid taxa. Tetraploid taxa of morphs 2 and 8 could have the genotypes X_2_X_2_X_4_Y, X_2_X_4_X_4_Y, or X_2_X_4_YY. In these cases, only genotype of X_2_X_4_X_4_Y, or X_2_X_4_YY and one probable evolutionary history of each genotype were illustrated to enhance the readability of Figure 6. It is also the most parsimonious scenario, having less presumed taxa. In this reticulate relationship (Figure 6), parts of the web are restricted to one geographic area, Hainan or Taiwan. This indicates that specific taxa were derived from specific progenitors (or alleles). For example, X_4_X_4_ occurs in Hainan and X_3_X_3_ in Taiwan.

The origin of apogamous diploids is an interesting example. Most apogamous diploids seem to come from hybrids between sexual diploid species and then acquired apogamy [53]. Given that apogamous diploids occurred earlier than apogamous polyploids, the apogamous diploids may be ancestors of the polyploids. However, “ploidy reduction” is an exception [59]. It was reported that the spores from one triploid *Dryopteris pacifica* (Nakai) Tagawa produced diploid and triploid gametophytes, and developed apogamous diploid and triploid sporophytes. The diploids and triploids in most taxa of the *P. cadieri* complex have different morphologies. Because no morphological differences were found in the offspring of the triploids in our cultures, “ploidy reduction” is unlikely to have been a factor in formation of the *P. cadieri* complex.

#### 2.2.4. Multiple Origins Supported by Genotypic, Geographic, and Phenological Divergence

Diverse genetic traits and taxa strongly suggest that the *P. cadieri* complex originated from multiple evolutionary events. Moreover, because taxa in the *P. cadieri* complex are apogamous [25,52], plants that differ genetically should be derived from different evolutionary events. Multiple events also explain the existence of the subtle genetic variation found within the three main clades of the cpDNA tree and within the five main *PgiC* clades (Figures 3 and 4).

Multiple hybridizations led to the geographic and phenological divergence of the *P. cadieri* complex. The distribution of several taxa limited to Hainan or Taiwan is due to specific genetic characters in Hainan or Taiwan (such as *PgiC* X_4_ and X_3_). Phenological divergence corresponded to genetic divergence, and to geographic distribution. For example, taxa in Taiwan with *PgiC* allele X_3_ were evergreen and only distributed in northern Taiwan. These correlations indicate these morphs could have arisen independently in different geographic areas and niches.

## 3. Experimental Section

### 3.1. Sampling and Phenological Studies

Samples of the *Pteris cadieri* complex, including *P. cadieri* and *P. grevilleana*, and of each morphological variant, were collected from Taiwan and Hainan, under evergreen broad leaf forest below 1000 m altitude (Supplementary S1). Because morphological variation in the *P. cadieri* complex exists among populations, among individuals in a population, and among fronds of an individual, the morphology of each plant was examined carefully during field work. At least three individuals, including a representative of several morphological variants, were collected from each population. Samples were collected from six populations in Hainan and 21 populations in Taiwan. In addition, the parental candidates, also having similar morphologies with *P. cadieri*, *P. cretica* L. (including four individuals with distinct morphologies, morphs A, B, C, and D) (Supplementary S1), *P. ensiformis* Burm., *P. kidoi* Sa. Kurata, *P. ryukyuensis* Tagawa, *P. morii* Masam., and *P. semipinnata* L. [18,22–24,55] were sampled. *Pteris dimidiata* Willd., *P. fauriei* Hieron., *P. longipinna* Hayata, *P. tokioi* Masam., *P. venusta* Kunze, and *P. wallichiana* J. Agardh were used as outgroups in phylogenetic analyses. Living plants were cultivated for one to five years in the greenhouse of the Taipei Botanical Garden for C-value measurements, phenological (evergreen or deciduous) studies, and morphological examinations. In the greenhouse, environmental effects on morphology were excluded. For phenological studies, the plants were determined to be either “evergreen” or “deciduous”. The “evergreen” plants produced new fronds all year, but “deciduous” plants lost all their fronds and did not produce new fronds from November to January. Voucher specimens were deposited at the herbarium (TAIF) of Taiwan Forestry Research Institute.

Type specimens of related taxa, including *Pteris cadieri* (in P), *P. dimorpha* (in K, MICH, and P), *P. hainanensis* (in IBSC and MICH), *P. grevilleana* (in B, E, and K), *P. plumbea* (in B, P, and TI), and *P. sintenensis* (in TI), were examined.

### 3.2. Ploidy Analysis and Reproductive Systems

Ploidy of each individual was determined by flow cytometry using fresh leaves from plants growing in the greenhouse. *Nicotiana tabacum* L. “Xanthi” (4X = 20.08 pg; [45]) was used as a calibration standard. Nuclei were extracted using CyStain PI absolute P kit (Partec, Münster, Germany). About 100 mm^2^ of fresh leaf tissue was chopped with a razor blade in 0.5 mL extraction buffer for 30–60 s, incubated for 10–15 min, filtered through a 50 mm nylon mesh (Partec, Münster, Germany), and processed in staining solution, containing RNase and propidium iodide (PI). Preparations were kept in the dark for 30 min. Ploidy was determined by FACScan (BD Technologies, Franklin Lakes, NJ, USA), using plants of known chromosome number as the control [25].

In most ferns, including the genus *Pteris*, sexual plants produce 64 spores per sporangium; apogamous plants produce 32 or fewer spores per sporangium [53,56,60,61]. Although it is known that *P. cadieri* and *P. grevilleana* are apogamous [25,52], to confirm the nature of the reproductive systems of plants in this study, the number of spores per sporangium was counted for each plant. Five mature sporangia were picked randomly from each plant. Spores, including shrunken ones, were counted, but debris was excluded.

### 3.3. Molecular Methods

Total genomic DNA was extracted from silica gel-dried leaves, following a modified CTAB method [62] or Plant Genomic DNA Mini Kit (Geneaid Biotech Ltd., Taipei, Taiwan).

Two chloroplast regions, the *rbcL* gene and *atpB-rbcL* spacer, and one nuclear region, the cytosolic phosphoglucose isomerase (*PgiC*) gene, were amplified and sequenced. Polymerase chain reaction amplification of the *rbcL* gene was performed with primers designed for this study (primer *rbcL*_PF; Table 2) and previous studies (primers *rbcL* F1F [48], *rbcL* F1379R [49], Table 2). The *atpB-rbcL* spacer was amplified by either of two pairs of primers (primers *atpB*_672 and *rbcL*_r49R [46], primers *atpB* 493F and *rbcL* r158R [47], Table 2). Polymerase chain reactions (PCR) were performed in a total volume of 25 μL containing 1× Ex buffer, 0.2 mM dNTPs, 0.06 μM of each primer, one unit of Taq polymerase (Genet Bio, Chungnam, Korea), and approximately 10 ng of template DNA. PCR amplifications were made using a T3 Thermocycler (Biometra, Göttingen) under the following conditions: denaturation for 5 min at 95 °C, 35 cycles of 45 s at 94 °C, annealing for 45 s at 54 °C (primers for *rbcL* gene) or 30 s at 57 °C (primers for *atpB-rbcL* spacer), and extension at 72 °C for 75 s (primers for *rbcL* gene) or 60 s (primers for *atpB-rbcL* spacer), with a final extension time of 8 min at 72 °C.

**Figure 7:**
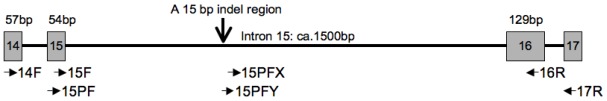
The positions of primers for *PgiC* gene from exon 14 to exon 17. Exons and introns are drawn to scale. Small arrows show positions and directions of the primers and the vertical arrow indicates the largest indel region.

For *PgiC*, the 14F and 16R primers [41] were initially used to amplify approximately 1.8 kb within exons 14, 15, and 16 in *Pteris*. Subsequently, a pair of new amplification primers, 15PF and 17R, were constructed for this study. Primer 15PF was located in intron 14 and exon 15, and primer 17R, designed from the cDNA of *Dryopteris* and *Arabidopsis* (NCBI), was located in exon 17. The amplified fragment includes 22 bp at the 3′ end of exon 15, two complete exons, two complete introns, and 35 bp from the 5′ end of exon 17 (Figure 7, Table 2). To avoid PCR bias (described below), two more forward primers, 15PFX and 15PFY, were designed. Amplifications were prepared in 25 μL reactions using 1× Ex buffer, 0.2 mM dNTPs, 0.08 μM of each primer, 0.6 units of Ex Taq polymerase (Genet Bio, Chungnam, Korea), 2% dimethylsulfoxide (DMSO), and approximately 20 ng of template DNA. PCR thermo cycling conditions were as follows: initial denaturation step of 95 °C for 5 min, followed by 35 cycles of denaturation at 94 °C for 45 s, annealing at 57 °C (primers 15PF and 17R), 54 °C (primers 15PFX and 17R) or 55 °C (primers 15PFY and 17R) for 45 s, and extension at 72 °C for 90 s, with a final extension at 72 °C for 8 min.

PCR products were purified using a Gel/PCR DNA Fragment Extraction Kit (Geneaid Biotech Ltd., Taipei, Taiwan). Some products were sequenced directly, while others were cloned into pGEM-T Easy vectors (Promega, Madison, WI, USA). Ligation, transformation, plating and selection of clones followed the instructions included with the kit. Plasmid DNA was purified using the High-Speed Plasmid Mini Kit (Geneaid Biotech Ltd., Taipei, Taiwan). Sequences were determined with an ABI 377 (Applied Biosystems Inc., Foster City, CA, USA) automated sequencer following the manufacturers protocols. A BLASTn search was conducted in GenBank to exclude contaminants. Previous studies indicated that there is a single copy of the *PgiC* gene [41], but it was not known whether this is true in *Pteris*. For an initial sampling of the *PgiC* gene from *Pteris* species, the PCR products were sequenced directly, and those that showed multiplicity or lacked clear base readings were cloned. In the *P. cadieri* complex, four to eight clones were sequenced from plants of different ploidy levels. We found that the number of *PgiC* gene alleles in each individual corresponded to that individual’s ploidy level. For example, a diploid plant had two alleles. On the other hand, some *Pteris* species had a single sequence and were presumed to be homozygous. Therefore, in *Pteris*, *PgiC* does appear to be a single-copy gene.

After initial cloning to design primers and develop PCR protocols for sequencing low-copy nuclear genes, initial analyses suggested the *PgiC* genes were effectively single-copy in diploids and that sequences in different individuals could be treated as ortholog.

We found marked PCR bias [50], in the PCR products from primers 15PF and 17R. In some samples, certain *PgiC* alleles were more difficult to isolate than other alleles. In order to gain all alleles in an individual, the largest indel region (15 bp) between exon 15 and 16 was used to design two, alternative forward primers, 15PFX and 15PFY, to primer 15PF (Figure 7, Table 2). Primer 15PFX was located in the insertion and primer 15PFY was located on both sides of the deletion. The PCR products were shorter than fragments amplified from the original primers, 14F and 16R, and most of them could be sequenced directly. If multiple *PgiC* sequences were amplified, cloning was used to isolate each allele. Clones were sequenced until two different types of alleles were captured more than once.

### 3.4. Phylogenetic Analysis

All DNA sequences were aligned using CLUSTAL X ver. 1.83 [63]. Subsequent manual corrections were carried out with BioEdit ver. 5.0.9. Maximum Parsimony analyses were performed with PAUP* ver. 4.0b10 [64] using a heuristic search algorithm, 100 random addition replicates, MULTREES option, no Steepest Descent, and tree-bisection reconnection (TBR) branch swapping. All characters were weighted equally and were unordered. In cpDNA sequences, indels were scored as missing data. We conducted an incongruence length difference test (ILD; [65]) to evaluate congruence between the *atpB-rbcL* spacer and the *rbcL* gene. The test was implemented in PAUP* with 1000 replicates of heuristic searches using parsimony informative characters. In *PgiC* data, ambiguously aligned regions were excluded and indels were scored as binary characters in phylogenetic analyses according to the method of “simple indel coding” [66] as implemented in the program GapCoder [67]. Branch support was evaluated by bootstrap analysis [68] with 1000 rounds of replication.

Maximum likelihood analyses were performed for cpDNA data using the program GARLI ver. 0.96 (Genetic Algorithm for Rapid Likelihood Inference, [69]). To ensure convergence to a similar topology and likelihood score, 10 independent runs were conducted using the automated stopping criterion or for up to 20,000 consecutive generations. To estimate the support for each node, 1000 bootstrap replicates were performed with the automated stopping criterion set at 10,000 generations. A 50% majority rule consensus tree of the 1000 bootstrap replicates from GARLI was then created using PAUP*.

Nuclear sequences, excluding indels, were analyzed, using a Bayesian inference with MrBayes ver. 3.1 [70]. The Tamura-Nei (TrN, [71] model of sequence evolution was chosen, based on results of the Akaike Information Criterion (AIC) as implemented in Modeltest ver. 3.7 [72]. The Akaike Information Criterion (AIC) was used to select the nucleotide substitution model. Among-site rate variation was allowed to follow a gamma distribution. Two runs with four chains each were run simultaneously for 10^6^ generations each run, with the temperature of the heated chains set to 0.2. The program Tracer ver.1.5 [73] was used to confirm that the parameters had converged. The initial 20% was discarded as burn-in. Chains were sampled every 100 generations and the respective trees written to a tree file. Convergence of the chains was assessed by stationary distribution of the likelihood value. After discarding burn-in samples, the remaining trees were loaded into PAUP* [64] to construct a majority rule consensus tree. Frequency values of the trees serve as estimates of the posterior probability of nodes.

## 4. Conclusions

This study identifies the maternal and paternal lineages of the *P. cadieri* complex. Morphological diversity of apogamous ferns is most likely due to multiple hybrid origins. Our understanding of the evolutionary history of the *P. cadieri* complex is crucial to further taxonomic and systematic studies. Scientific names could not be assigned to all eight morphs. Moreover, taxonomic treatment of morphologically similar but genetically distinct taxa presents an interesting challenge [74]. For morph 8, the molecular data indicate that tetraploids are genetically distinct from diploids and triploids, and tetraploids occur only on Hainan. However, they are difficult to distinguish morphologically. In contrast, plants with the same genetic character exhibiting distinct morphologies also result in taxonomic problem. For example, morphs 3, 4, and 7 shared the same *PgiC* genotypes, but exhibited distinctly different frond morphologies. Similarly, this kind of epiallelic variation has also been found in other plant species [75–77]. In addition, the DNA marker, the *PgiC* gene, could have limitations and might not differ in these morphs.

In a species complex with a complicated evolutionary history, no single method can be used to delimit a taxon. This study provides an example of the importance of reticulate evolution in fern speciation. The ancestral taxa of the *P. cadieri* complex apparently repeatedly and reciprocally crossed with one another. Following each hybridization event, apogamy shut down gene flow, genetically isolating each new taxon. Recurrent hybridization led to complex variation in the morphology and ploidy of taxa in the *P. cadieri* complex. It is likely that many hybrid taxa became extinct, while others persist to the present day and now comprise the *P. cadieri* species complex as we know it. Lost lineages make it difficult to reconstruct the complete evolutionary history of the *P. cadieri* complex. Because the *P. cadieri* complex is widely distributed in eastern and southern Asia, hybridization could have occurred extensively and independently in populations in other regions.

## Supplementary Information



## Figures and Tables

**Table 1:** Morphological characteristics for identifying taxa in the *Pteris cadieri* complex.

Morph type	Ploidy level	Fertile blades	Sterile blades	Scales
Morph 1	Diploid	Pedate or pinnate	Pedate, basal pinna straight or incurved	Wide and dark brown center
Morph 2	Diploid, tetraploid	Pedate or pinnate, sometimes with digital projects	Pedate, basal pinna straight or incurved	Narrow and dark brown center
Morph 3	Diploid	Pinnate, sometimes with digital projects	Pinnate, sometimes with digital projects, basal pinna straight	Wide and dark brown center
Morph 4	Diploid	Pinnate, sometimes with digital projects	Bipinnatifid, basal pinna straight	Wide and dark brown center
Morph 5	Triploid	Pedate, sometimes with digital projects	Bipinnatifid, basal pinna incurved	Wide and dark brown center
Morph 6	Triploid	Irregularly bipinnatifid	Bipinnatifid, basal pinna incurved	Wide and dark brown center
Morph 7	Diploid	Almost regularly bipinnatifid	Bipinnatifd, basal pinna straight	Wide and dark brown center
Morph 8	Diploid, triploid, tetraploid	Bipinnatifid	Bipinnatifid, basal pinna straight or incurved	Wide and dark brown center

**Table 2:** PCR primers used in this study.

	Primer for Chloroplast DNA	

Primer	Primer sequence (5′→3′)	Origin
*atpB*_672	CAC TSA GAG GRG CTC CCG TAT CAA	[46]
*rbcL*_r49R	CAC CAG CTT TGA ATC CAA CAC TTG C	[46]
*atpB* 493F	CGA CGA TAC GGR GCC AAA AGA TCC	[47]
*rbcL* r158R	AAG ATT CCG CAG CTA CTG CAG CTC C	[47]
*rbcL* F1F	ATG TCA CCA CAA ACA GAA ACT AAA GCA AGT	[48]
*rbcL* F1379R	TCA CAA GCA GCA GCT AGT TCA GGA CTC	[49]
*rbcL*_PF	TAA GTA TCG TGY GGA GGT TRA ATC A	This study

	**Primer for*PgiC***	

**Primer**	**Primer sequence (5′→3′)**	**Origin**

14F	GTG CTT CTG GGT CTT TTG AGT G	[41]
16R	GTT GTC CAT TAG TTC CAG GTT CCC C	[41]
15PF	CAAATCCTTTCTTGCAATAGGC	This study
17R	GAA ATCAC ATGGA ATAAC ACGTCC	This study
15PFX	CAAGT ATACC TCTTC TTGAC AG	This study
15PFY	CAG CAA GTA TAA CAA AAA CTC GC	This study

**Table 3:** Dataset and parsimony-based tree statistics for three DNA data. Including *atpB-rbcL* spacer & *rbcL* gene, *PgiC* gene by primers 15PF and 17R, and *PgiC* gene by forward primers 15PFX/15PFY and reverse primer 17R.

	*atpB-rbcL* spacer & *rbcL* gene	*PgiC* gene (primers 15PF and 17R)	*PgiC* gene (primers 15PFX/15PFY and 17R)
No. sequence	57	151	191
Aligned length (bp)	2126	1794	1090
Characters included (bp)	2091–2119	1431–1705	786–1050
Variable characters (bp)	227	559	399
Parsimony informative characters (bp)	98	306 (50 binary characters produced by coding indels)	200 (41 binary characters produced by coding indels)
Obtained trees	322	4128	1145
Tree length	300	774	578
CI	0.777	0.764	0.739
RI	0.930	0.929	0.937
