# Supplementary material for: Extreme Multiple Reticulate Origins of the Pteris cadieri Complex (Pteridaceae)
Source: Int J Mol Sci. 2012 Apr 10;13(4):4523–44. doi: 10.3390/ijms13044523 (PMC3344230; doi:10.3390/ijms13044523)

Supplementary Materials

S1. Specimen information and GenBank accession number. Voucher specimens were deposited in the herbarium (TAIF) of Taiwan Forest Research Institute. (NA = not available). **Taxon**—specimen code, ploid level, voucher specimen, locality, Genbank accession numbers for *atpB-rbcL* spacer + *rbcL* gene, *PgiC* gene, (*PgiC* gene).

"*P. cadieri*" = morph 1—cp15, 2X, *Chao742*, Chingjenhu, Keelung City, Taiwan, HM582607, HM588775, HM588776; cp95, *Chao896*, Mt. Wanlite, Pingtung County, Taiwan, HM582653, NA; cp95, *Chao896*, Mt. Wanlite, Pingtung County, Taiwan, NA, JQ247254, JQ247255; cp147, 2X, *P. F. Lu s.n. 20060317*, Nanjenshan, Pingtung County, Taiwan, HM582605, HM588817, HM588818, HM588819, HM588820, HM588821, HM588822; cp155, 2X, *Chao1042*, Lienhuachih, Nantou County, Taiwan, HM582606, HM588826, HM588827, JQ247257, JQ247258; cp187, 2X, *Chao1067*, Mt. Wanlite, Pingtung County, Taiwan, HM582611, JQ247263, JQ247264; cp232, 2X, *Chao1191*, Wuzhishan National Nature Reserve, Hainan, HM582617, HM588844, HM588845; cp233, 2X, *Chao1192*, Wuzhishan National Nature Reserve, Hainan, HM582618, NA; cp242, 2X, *Chao1210*, Wuzhishan National Nature Reserve, Hainan, HM582620, JQ247277, JQ247278; cp245, 2X, *Chao1219*, Wuzhishan National Nature Reserve, Hainan, HM582621, HM588846, HM588847, HM588848, JQ247279, JQ247280; cp271, 2X, *Chao1248*, Shiqing, Diaoluosha, Hainan, HM582626, JQ247287, JQ247288; cp285, 2X, *Chao1264*, Baishuiling, Diaoluosha, Hainan, HM582632, JQ247290, JQ247291; cp386, 2X, *P. F. Lu s.n. 20080223-7*, Fulong, Taipei County, Taiwan, NA, HM588892, HM588893, HM588894, JQ247295; cp424, 2X, *L. Y. Kuo s.n. 20080815-10*, Nanjenshan, Pingtung County, Taiwan, NA, JQ247298, JQ247299; cp431, 2X, *P. F. Lu s.n. 20080831-1*, From Taiyang Pond to Leng Pond, Ilan County, Taiwan, NA, HM588898, HM588899, JQ247300; cp440, 2X, *P. F. Lu s.n. 20081004-2*, Shuangsi, Taipei County, Taiwan, HM582639, HM588902, HM588903, HM588904, HM588905; cp486, NA, *Y. H. Chang s.n. 20090213-1*, Feitsui Reservoir, Taipei County, Taiwan, HM582642, HM588908, HM588909, HM588910; cp491, 2X, *Mo1804*, Sanhsia, Taipei County, Taiwan, HM582643, HM588911, HM588912, HM588913, JQ247306.

"*P. cadieri*" = morph 2—cp231, 2X, *Chao1179*, Exianling, Hainan, HM582616, HM588843, JQ247275, JQ247276; cp251, 4X, *Chao1225*, Shuiman, Wuzhishan National Nature Reserve, Hainan, HM582622, HM588849, HM588850, HM588851, HM588852; cp256, 4X, *Chao1232*, Shuiman, Wuzhishan National Nature Reserve, Hainan, HM582623, HM588853, HM588854, HM588855, HM588856, HM588857, HM588858, HM588859; cp262, 4X, *Chao1239*, Shiqing, Diaoluosha, Hainan, HM582625, HM588862, HM588863, HM588864, HM588865, HM588866, JQ247285, JQ247286; cp281, 4X, *Chao1260*, Baishuiling, Diaoluosha, Hainan, HM582630, HM588877, HM588878, HM588879, HM588880, HM588881; cp283, 4X, *Chao1262*, Baishuiling, Diaoluosha, Hainan, HM582631, HM588882, HM588883, HM588884, HM588885, HM588886, JQ247289.

"*P. cadieri*" = morph 3—cp29, 2X, *Chao758*, Taoyuan Valley Trail, Taipei County, Taiwan, HM582633, HM588781, HM588782, HM588783, HM588784, JQ247247; cp40, 2X, *Chao764*, Nangang, Taipei City, Taiwan, HM582637, HM588785, HM588786, JQ247248; cp46, 2X, *Chao743*, Chingjenhu, Keelung City, Taiwan, NA, JQ247249, JQ247250; cp258, 2X, *Chao1235*,

Shuiman, Wuzhishan National Nature Reserve, Hainan, NA, JQ247281, JQ247282; cp385, 2X, *P. F. Lu s.n. 20080223-6*, Fulong, Taipei County, Taiwan, HM582634, HM588887, HM588888, HM588889, HM588890, HM588891, JQ247294; cp436, 2X, *P. F. Lu s.n. 20081005-2*, Tahsi, Taoyuan County, Taiwan, HM582638, HM588900, HM588901; cp480, 2X, *C. W. Chen s.n. 20090205-2*, Yuemeishan, Hualien County, Taiwan, HM582641, HM588906, HM588907.

"*P. cadieri*" = morph 4—cp6, 2X, *Chao735*, Shuangsi, Taipei County, Taiwan, HM582648, HM588768, HM588769; cp10, 2X, *Chao739*, Shuangsi, Taipei County, Taiwan, HM582599, HM588770, HM588771, HM588772, HM588773, HM588774,; cp180, 2X, *Chao1062*, Hsinshan-menghu, Taipei County, Taiwan, NA, JQ247261, JQ247262; cp211, 3X, *Chao1153*, Fushan, Ilan County, Taiwan, HM582614, HM588833, HM588834, JQ247270; cp226, 2X, *Chao1174*, Exianling, Hainan, NA, HM588835, JQ247271, JQ247272; cp227, 2X, *Chao1175*, Exianling, Hainan, NA, HM588836, HM588837, JQ247273, JQ247274; cp259, 2X, *Chao1236*, Shuiman, Wuzhishan National Nature Reserve, Hainan, HM582624, HM588860, HM588861, JQ247283, JQ247284.

"*P. cadieri*" = morph 5—cp71, 3X, *Mo20050925-07*, Chungho, Taipei County, Taiwan, HM582649, HM588796, HM588797, HM588798, HM588799, HM588700, JQ247253.

"*P. cadieri*" = morph 6—cp3, 3X, *Chao732*, Shuangsi, Taipei County, Taiwan, HM582636, HM588766, HM588767, JQ247246; cp468, 3X, *P. F. Lu s.n. 20090103-1*, Chiuchiungkenshan, Taipei County, Taiwan, HM582640, JQ247301, JQ247302, JQ247303, JQ247304, JQ247305.

"*P. cadieri*" = morph 7—cp18, 2X, *Chao746*, Taoyuan Valley Trail, Taipei County, Taiwan, HM582612, HM588777, HM588778, HM588779, HM588780; cp60, 2X, *P. F. Lu 10431*, Wulai Township, Taipei County, Taiwan, HM582646, JQ247251, JQ247252.

"*P. cadieri*" = morph 8—cp51, 2X, *Chao770*, Nanjenshan, Pingtung County, Taiwan, HM582644, HM588787, HM588788, HM588789, HM588790, HM588791; cp123, 3X, *P. F. Lu 11381*, Chingjenhu, Keelung City, Taiwan, HM582602, HM588815, HM588816; cp154, 2X, *Chao1041*, Lienhuachih, Nantou County, Taiwan, NA, HM588823, HM588824, HM588825, JQ247256; cp171, 2X, *Chao721*, Kaoshihchiuchih, Pingtung County, Taiwan, HM582609, HM588828, HM588829, HM588830, JQ247259, JQ247260; cp188, 2X, *Chao1068*, Mt. Wanlite, Pingtung County, Taiwan, NA, JQ247265, JQ247266; cp190, 2X, *Chao1070*, Mt. Wanlite, Pingtung County, Taiwan, NA, JQ247267, JQ247268; cp197, 3X, *Chao1075*, Gupoliao River, Taipei County, Taiwan, NA, HM588831, JQ247269; cp239, NA, *Chao1205*, Wuzhishan National Nature Reserve, Hainan, HM582619, NA; cp273, 4X, *Chao1250*, Shiqing, Diaoluosha, Hainan, HM582627, HM588867, HM588868, HM588869, HM588870, HM588871, HM588872, HM588873, HM588874, HM588875, HM588876; cp274, 4X, *Chao1251*, Shiqing, Diaoluosha, Hainan, HM582628, NA; cp275, NA, *Chao1252*, Shiqing, Diaoluosha, Hainan, HM582629, NA; cp358, 2X, *L. Y. Kuo s.n. 20071209-1*, Luliao river, Pingtung County, Taiwan, NA, JQ247292, JQ247293; cp406, 2X, *Y. H. Chang s.n. 20080710*, Shouchia, Pingtung County, Taiwan, NA, JQ247296, JQ247297.

***P. cretica*_morph A (fronds length <30 cm; denticulate pinna)**—cp54, NA, *Chao792*, Wulingshan Nantou County, Taiwan, HM582645, HM588792, HM588793, HM588794, HM588795.

***P. cretica*_morph B (fronds length >30 cm; denticulate pinna)**—cp96, NA, *Chao910*, Hsinkangshan, Hualien County, Taiwan, HM582654, HM588807, HM588808, HM588809.

***P. cretica*_morph C (fronds length >30 cm; serrate pinna)**—cp389, NA, *Chao1428*, Jianshi, Hsinchu County, Taiwan, HM582635, HM588895, HM588896, HM588897.

***P. cretica*_morph D (fronds length <30 cm; serrate pinna)**—cp63, NA, *Chao855*, Kaotingshan, Kaohsiung County, Taiwan, HM582647, NA.

P. dimidiata—cp174, NA, *Chao725*, Kaoshihchiuchih, Pingtung County, Taiwan, HM582610, NA.

P. ensiformis—cp87, NA, *Chao888*, Taipei Botanical Garden, Taipei City, Taiwan, HM582652, NA.

P. fauriei—N52, NA, *Y. M. Huang s.n. 20030513-31*, Chialeshui, Pingtung County, Taiwan, HM582608, JQ247232, JQ247233

P. kidoi—cp198, NA, *Dai1917*, Shakadang Trail, Hualien County, Taiwan, HM582613, HM588832.

P. longipinna—cp106, NA, *Chao953*, Taimali, Taitung County, Taiwan, HM582603, HM588810, HM588811, HM588812.

P. morii—cp228, NA, *Chao1176*, Exianling, Hainan, HM582615, HM588838; cp229, NA, *Chao1177*, Exianling, Hainan, NA, HM588839, HM588840, HM588841, HM588842.

P. ryukyuensis—cp121, NA, *P. F. Lu 11375*, Chingjenhu, Keelung City, Taiwan, HM582601, HM588813, HM588814.

P. semipinnata—cp143, NA, *P. F. Lu 11384*, Guguan, Taichung County, Taiwan, HM582604, NA.

P. tokioi—cp100, NA, *Chao935*, Taimali, Taitung County, Taiwan, HM582600, JQ247237, JQ247238.

P. venusta—cp79, NA, *Chao873*, Chiweishan, Kaohsiung County, Taiwan, HM582650, HM588801, HM588802, HM588803; cp80, NA, *Chao881*, Chiweishan, Kaohsiung County, Taiwan, HM582651, HM588804, HM588805, HM588806.

P. wallichiana—cp99, NA, *Chao932*, Taimali, Taitung County, Taiwan, HM582655, JQ247239.

Figure S2. One of the maximum parsimony trees of the *Pteris cadieri* complex and other *Pteris* species. Primers 15PFX/15PFY and 17R of *PgiC* gene were used. The grouping of alleles X_1, X_2, X_3, X_4 and Y correspond to the *PgiC* trees based on primers 15PF and 17R (Figure 4). However, some branches have supporting bootstrap values less than 50%. The number following the specimen code indicates the cloning sample (see Supplementary S1). If a specimen code is not followed by a cloning sample number, the sample was sequenced directly.

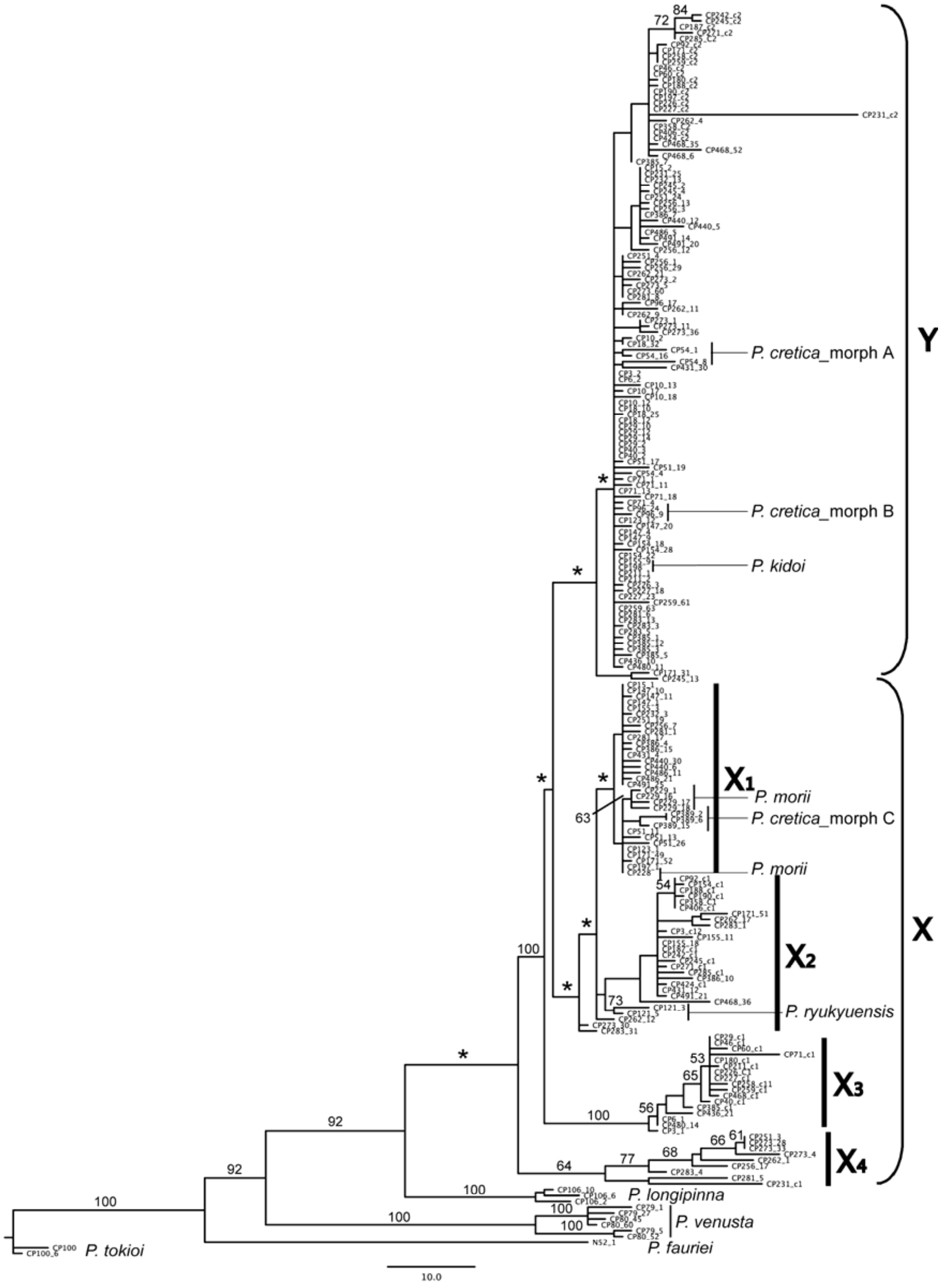

Supplement: Supplementary file 1 [file ijms-13-04523-s001.pdf]
